# Biochemical Characterization of *Mycobacterium tuberculosis* DNA Repair Enzymes – Nfo, XthA and Nei2

**DOI:** 10.5195/cajgh.2013.107

**Published:** 2014-01-24

**Authors:** Sailau Abeldenov, Murat Saparbayev, Bekbolat Khassenov

**Affiliations:** 1 National Center for Biotechnology, Astana, Kazakhstan; 2 Institut Gustave Roussy, Villejuif, France

**Keywords:** tuberculosis, DNA sequencing, DNA repair enzymes

## Abstract

**Introduction:**

Tuberculosis (TB) is a human disease caused by *Mycobacterium tuberculosis (*Mtb*)*. Treatment of TB requires long-term courses of multi-drug therapies to eliminate subpopulations of bacteria, which sometimes persist against antibiotics. Therefore, understanding of the mechanism of Mtb antibiotic-resistance is extremely important.

During infection, Mtb overcomes a variety of body defense mechanisms, including treatment with the reactive species of oxygen and nitrogen. The bases in DNA molecule are susceptible to the damages caused by reactive forms of intermediate compounds of oxygen and nitrogen. Most of this damage is repaired by the base excision repair (BER) pathway. In this study, we aimed to biochemically characterize three Mtb DNA repair enzymes of BER pathway.

**Methods:**

*XthA*, *nfo,* and *nei* genes were identified in mycobacteria by homology search of genomic sequences available in the GenBank database. We used standard methods of genetic engineering to clone and sequence Mtb genes, which coded Nfo, XthA and Nei2 repair enzymes. The protein products of Mtb genes were expressed and purified in *Escherichia coli* using affinity tags. The enzymatic activity of purified Nfo, XthA, and Nei2 proteins were measured using radioactively labeled DNA substrates containing various modified residues.

**Results:**

The genes *end* (Rv0670), *xthA* (Rv0427c), and *nei* (Rv3297) were PCR amplified using genomic DNA of Mtb H37Rv with primers that contain specific restriction sites. The amplified products were inserted into pET28c(+) expression vector in such a way that the recombinant proteins contain C-terminal histidine tags. The plasmid constructs were verified by sequencing and then transformed into the *Escherichia coli* BL21 (DE3) strain. Purification of recombinant proteins was performed using Ni^2+^ ions immobilized affinity column, coupled with the fast performance liquid chromatography machine AKTA. Identification of the isolated proteins was performed by protein mass spectrometry by ion trap tandem MS/MS on nLC-ESI-Ion-Trap platform. Biochemical characterization of DNA repair protein-catalyzed activity was carried out by measuring apurinic/apyrimidinic endonuclease, DNA glycosylase, exonuclease, and 3′-repair diesterase functions. In addition, effect of the opposite base and the influence of metal ion cofactors were measured.

**Conclusion:**

Results of the ongoing study will help us define the role of DNA repair enzymes in the emergence of mutations in the mycobacterial genome and, possibly, the origins of multi-drug resistance in mycobacteria.

